# Quantitative proteomic analyses of two soybean low phytic acid mutants to identify the genes associated with seed field emergence

**DOI:** 10.1186/s12870-019-2201-4

**Published:** 2019-12-19

**Authors:** Xiaomin Yu, Hangxia Jin, Xujun Fu, Qinghua Yang, Fengjie Yuan

**Affiliations:** Institute of Crop and Nuclear Technology Utilization, Zhejiang Academy of Agricultural Sciences, Hangzhou, 310021 China

**Keywords:** Soybean, Low phytic acid, Field emergence, Germination, Proteomics

## Abstract

**Background:**

Seed germination is essential to crop growth and development, and ultimately affects its harvest. It is difficult to breed soybeans low in phytic acid with a higher seed field emergence. Although additional management and selection could overcome the phytate reduction, the mechanisms of seed germination remain unknown.

**Results:**

A comparative proteomic analysis was conducted between two low phytic acid (LPA) soybean mutants (TW-1-M and TW-1), both of which had a deletion of 2 bp in the *GmMIPS1* gene. However, the TW-1 seeds showed a significantly lower field emergence compared to the TW-1-M. There were 282 differentially accumulated proteins (DAPs) identified between two mutants at the three stages. Among these DAPs, 80 were down-accumulated and 202 were up-accumulated. Bioinformatic analysis showed that the identified proteins were related to functional categories of oxidation reduction, response to stimulus and stress, dormancy and germination processes and catalytic activity. KEGG analysis showed that these DAPs were mainly involved in energy metabolism and anti-stress pathways. Based upon the conjoint analysis of DAPs with the differentially expressed genes (DEGs) previously published among three germination stages in two LPA mutants, 30 shared DAPs/DEGs were identified with different patterns, including plant seed protein, beta-amylase, protein disulfide-isomerase, disease resistance protein, pyrophosphate-fructose 6-phosphate 1-phosphotransferase, cysteine proteinase inhibitor, non-specific lipid-transfer protein, phosphoenolpyruvate carboxylase and acyl-coenzyme A oxidase.

**Conclusions:**

Seed germination is a very complex process in LPA soybean mutants. The TW-1-M and TW-1 showed many DAPs involved in seed germination. The differential accumulation of these proteins could result in the difference of seed field emergence between the two mutants. The high germination rate in the TW-1-M might be strongly attributed to reactive oxygen species-related and plant hormone-related genes. All these findings would help us further explore the germination mechanisms in LPA crops.

## Background

Soybean is one of the most important commercial crops planted all over the world due to its excellent source of protein, oil and many functional substances, such as lecithin, isoflavon and saponin [1]. However, soybean seeds are susceptible to internal damage during their germination and development stages due to their high content of protein and oil. Therefore, it is essential to plant seeds with high-emergence rates to obtain a satisfactory yield. The germination, vigor and emergence rates could be significantly reduced under unfavorable environmental conditions (such as drought, freezing and salt stress) during seed germination and development stages [1–4] or with the changes in seed chemical components (such as phytic acid content and fatty acid composition) [5–7]. Thus, seed germination trait should be fully considered during the improvement of ecological and nutritional traits. There were many studies that focused mainly on the outer environmental damage [2–4]. However, the internal damage of seeds has rarely been focused on according to the changes of nutritional and anti-nutritional factors [5, 7].

It will be beneficial to lower seed phytic acid (phytate) content and thus improve nutritional traits in crop seeds and decrease phosphorus level in water. Therefore, it is needed to generate crops with disrupted phytate synthesis during seed development [8–10]. During the last three decades, many low phytate crops were developed by using different techniques. All these crops showed significant decrease in phytate contents compared to wild type. Unfortunately, some of them also exhibited a decline in seed germination ability, such as soybean and rice [5, 7, 11, 12]. This unfavorable trait hindered the application of these low phytate germplasms in crop breeding program. Therefore, many efforts are still needed to improve the germination trait of low phytate crop lines. We developed a low phytic acid (LPA) soybean mutant, Gm-lpa-TW-1 (TW-1), with a 2-bp deletion of the *GmMIP1* gene. The TW-1 mutant exhibited a low rate of field emergence and its germination rate reduced rapidly during seed storage [13]. The progenies of cross-breeding TW-1 and other commercial soybean varieties also showed a lower seeds field emergence. The F_1_ seeds had normal seed field emergence. For F_2_ individuals and F_3_-F_5_ lines, seed germination rates were more or less impacted by phytate content and the level of influence depended on the different genetic background (Data not shown). It is difficult to predict which kind of varieties could be used to improve the traits of seed field emergence of TW-1. Fortunately, a natural mutated line, Gm-lpa-TW-1-M (TW-1-M), was found with a higher field emergence compared with the other TW-1 lines. The phytate contents of TW-1 and TW-1-M were 9.5 and 10.5 mg/g respectively and much lower than their parent Taiwan-75 (14.5 mg/g). Although the TW-1-M mutant and the TW-1 mutant had the same mutation site according to the sequencing analysis of the *GmMIPS1* gene, the TW-1-M exhibited significantly enhanced seed germination ability and high seed emergence rate, despite of them very similar genetic background. The progenies of TW-1-M with other commercial soybean varieties also had normal seed field emergence. Therefore, it is worthwhile to understand the mechanism of this new LPA soybean line with high-germination rates.

Large-scale gene expression analysis could provide insights into both the environmental and genetic influences on seed germination. A comparative transcript analysis was performed between the TW-1-M and TW-1 at seed germination [14]. There were approximately 3900–9200 differentially expressed genes (DEGs) identified at each stage between these two mutants [14]. The numbers of up-regulated and down-regulated genes were comparable between the TW-1-M and TW-1. In comparison with the TW-1, the TW-1-M mutant exhibited a lower level of gene expression in ethylene synthesis, but a higher level of gene expression in stress response, energy metabolism, anti-oxidation and GA biosynthesis processes during seed germination. The TW-1-M exhibited a higher germination rate, which might be strongly due to the expression diversification of antioxidation-related and hormone-related genes [14].

Improving LPA seed germination requires the modification of gene expression, whereas the encoded proteins are required to determine gene function by proteomics. Several proteomic methods have broadened knowledge to explore the regulating mechanisms during seed germination [15, 16]. However, proteomic mechanisms are still little known according to the low germination in LPA soybean until now. While the genetic analysis of LPA soybean has been carried out to investigate seed germination, a proteomic method could further uncover the features of LPA trait at the protein level. Hence, this study was conducted to evaluate a great amount of protein information and then conjoint analysis of differentially accumulated proteins and differentially expressed genes. The objective was to investigate seed germination and identify candidate genes involved in seed germination of LPA soybean. Here, we applied isobaric tag for relative and absolute quantification (iTRAQ) technology to investigate proteome in seeds of two LPA soybean mutants at different germination stages.

## Results

### Seed germination of two LPA mutants

The seeds of the TW-1-M, TW-1 and Taiwan-75 (their parent) were tested with both accelerated aging and warm germination treatments. Seed germination rates were analyzed by measuring the number and speed of germinating seeds. Significant differences were detected in the germination speed between the two mutants with both accelerated aging and warm germination treatments. It took about 72 h for TW-1-M to get the highest germination percentage, but TW-1 need about 96 h to reach the same level. The germination speeds of Taiwan-75 and TW-1 were comparable in both the treatments.

**Fig. 1:**
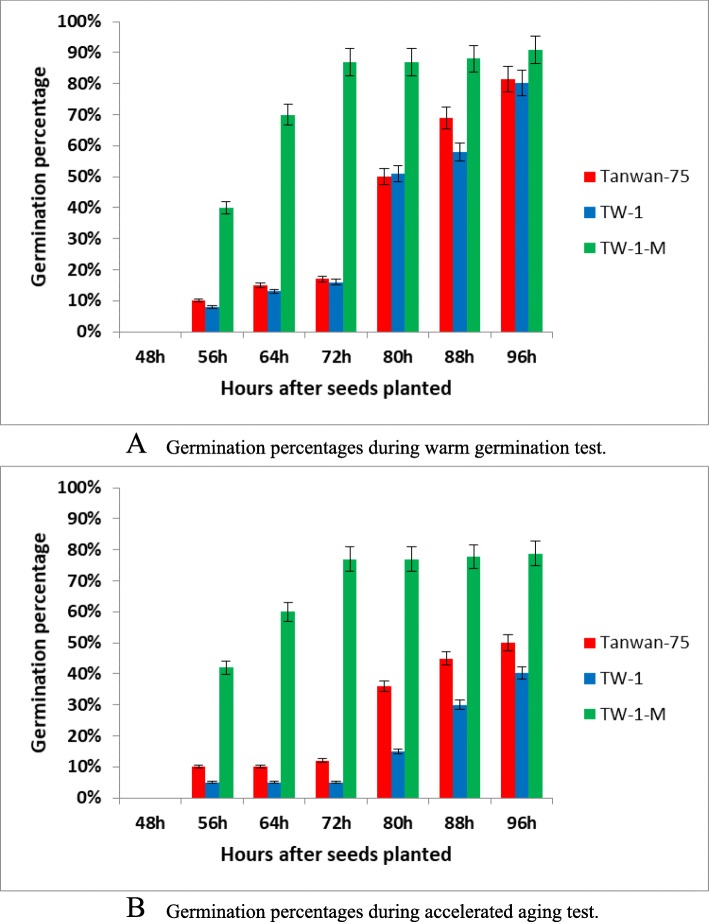
Germination percentages of two LPA mutants and their wild-type parent. **a** germination percentages during warm germination test; **b** germination percentages during accelerated aging test. In both the warm germination test and accelerating aged test, the mutant TW-1-M performed well, with a high germination percentage (more than 80%) and speed, compared with the TW-1 and Taiwan-75

In the warm germination test, TW-1 and Taiwan-75 had a similar germination percentage as about 80%, but the percentage of TW-1-M was about 90%, higher than TW-1 and Taiwan-75 (Fig. 1a). In the accelerated aging test, the germination percentages of TW-1 and Taiwan-75 were 40 and 50%, respectively, but TW-1-M performed well at about 78% (Fig. 1b). Therefore, the seed germination of TW-1-M was significantly better than those of Taiwan-75 and TW-1. The TW-1-M mutant exhibited better storage stability and germination trait compared with the TW-1. The field emergence of the TW-1-M, TW-1 and Taiwan-75 also confirmed this result (Data not shown).

### Protein sequence coverage, protein identification and data analysis

To characterize protein accumulation profiles during seed germination, the high throughput proteome analyses of seedling libraries were performed using an iTRAQ-based quantitative proteomics approach. Each sample of TW-1-M-1, TW-1-1, TW-1-M-2, TW-1-2, TW-1-M-3 and TW-1-3 was analyzed in a batch with three replicates. A mean total of 320,584 spectrum were generated in the iTRAQ experiment, a mean of 94,932 spectrum identified were matched to the known spectrum. The spectrum identification rates of the three batches were 27.65, 32.39 and 28.57%, respectively. The mean peptide identification of three batches was 10,146. The numbers of the identified proteins containing at least two unique peptides in the three batches were 1205, 1297 and 1234, respectively (Table 1). The numbers of the identified proteins in the three batches were 1694, 1788 and 1803, respectively, and a total of 877 proteins were detected in all the three batches (Additional file 2: Figure S1).

**Table 1:** The identified proteins in the three batches

Sample batch	Total spectrum	Spectrum identification	Spectrum identification rate	Peptide identification	Protein identification	Protein identification (2unique peptides)
Batch I	311697	86187	27.65%	9506	1694	1205
Batch II	337657	109364	32.39%	10885	1788	1297
Batch III	312399	89246	28.57%	10048	1803	1234
Mean	320584	94932	29.54%	10146	1762	1245

### Identification of DAPs by iTRAQ

**Fig. 2:**
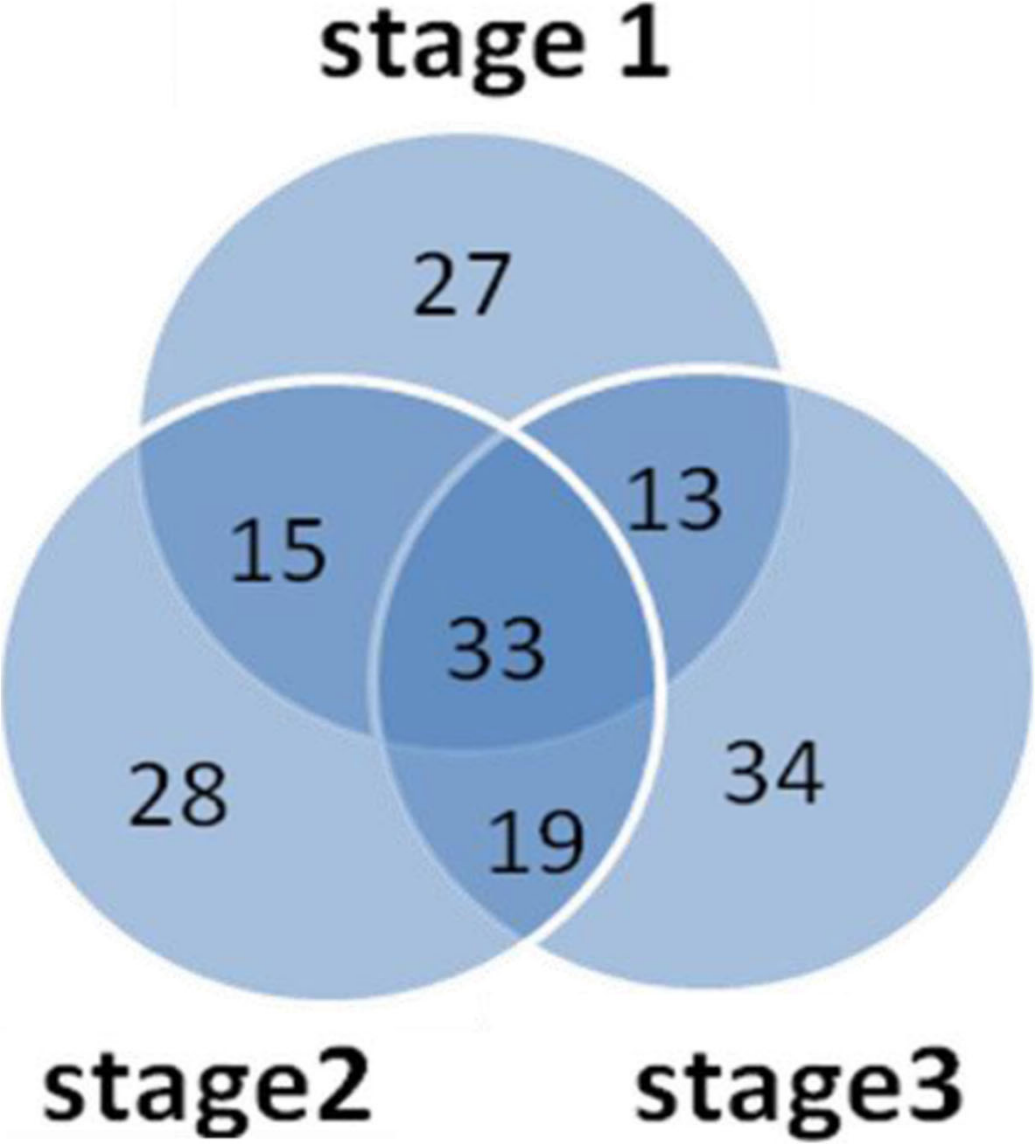
Venn diagrams showing the overlapping of DAPs in three germination stages of two comparing LPA soybean mutants

To identify the differentially accumulated proteins (DAPs) during seed germination, we performed pairwise comparisons of iTRAQ data between TW-1-M and TW-1 at each stage. To analyze the significance of the accumulated protein differences, two criteria were applied: fold change > 1.2 and *P*-value < 0.05. We found that totally 282 DAPs were identified between the two mutants at each stage. Between TW-1-M-1 and TW-1-1 (TW-1-M-1 vs. TW-1-1), 66 proteins were down-accumulated and 22 proteins were up-accumulated. There were 64 proteins down-accumulated and 31 proteins up-accumulated in TW-1-2 compared to TW-1-M-2 (TW-1-M-2 vs. TW-1-2). In addition, 72 proteins were down-accumulated and 27 proteins were up-accumulated between TW-1-3 and TW-1-M-3 (TW-1-M-3 vs. TW-1-3). Among three different germination stages, the number of down-accumulated proteins was always more than the up-accumulated protein number, and the total number of DAPs increased along with the germination stage in depth (Table 2). Furthermore, we also compared the DAPs in pairwise among three different germination stages, there are 33 common DAPs through the whole germination process, and 27, 28, 34 specific DAPs in three stages (Fig. 2).

**Table 2:** The significantly accumulated protein between the two samples

Comparison	Up-accumulated proteins	Down-accumulated proteins	Total up/down-accumulated proteins
TW-1-M-1/TW-1-1	22	66	88
TW-1-M-2/TW-1-2	31	64	95
TW-1-M-3/TW-1-3	27	72	99

### Bioinformatics analysis of DAPs identified by iTRAQ

In this study, most of the DAPs from three different stages could be divided into the three categories: biological process, cellular component and molecular function. With respect to biological process, DAPs were mainly included in metabolic process, response to stimulus, cellular process and developmental process [14]. Under cellular component, most DAPs were involved in the categories of cell, cell part, and organelle. With regard to molecular function, the major DAP categories were catalytic activity, binding, and nutrient reservoir activity. A new category, antioxidant activity appeared at the TW-1-M-3 vs. TW-1-3 database, and five proteins in nutrient reservoir activity category were all up-accumulated. All these categories contained up/down accumulated proteins which were more than 10% (Additional file 2: Figure S2).

A KEGG pathway analysis was further conducted to elucidate DAP biological functions. The DAPs in 84 pathways were identified at the TW-1-M-1 vs. TW-1-1 stage. The main regulatory pathways (without directions) with the most protein numbers were metabolic pathways (22 proteins), microbial metabolism in diverse environments (14 proteins), biosynthesis of secondary metabolites (11 proteins) and protein processing in endoplasmic reticulum (10 proteins), glycolysis/gluconeogenesis (7 proteins), linoleic acid metabolism (6 proteins), butanoate metabolism (5 proteins), chloroalkane and chloroalkene degradation (5 proteins), starch and sucrose metabolism (5 proteins), fructose and mannose metabolism (5 proteins). There were 61 pathways responsible for the differences at the TW-1-M-2 vs. TW-1-2 stage. Among these pathways, metabolic pathways (20 proteins), microbial metabolism in diverse environments (10 proteins), biosynthesis of secondary metabolites (8 proteins), protein processing in endoplasmic reticulum (6 proteins), linoleic acid metabolism (5 proteins), starch and sucrose metabolism (5 proteins) and glycolysis/gluconeogenesis (5 proteins) have the most protein numbers. There were 77 pathways involved in the comparison between TW-1-M-3 and TW-1-3, the pathways which have the most protein numbers were metabolic pathways (23 proteins), microbial metabolism in diverse environments (13 proteins), biosynthesis of secondary metabolites (13 proteins), glycolysis/gluconeogenesis (7 proteins), alpha-Linolenic acid metabolism (6 proteins), protein processing in endoplasmic reticulum (6 proteins) and linoleic acid metabolism (5 proteins) (Additional file 1). The KEGG results suggested that most of DAPs were involved in response to different environment and energy metabolism (including carbohydrate, protein and fat) pathway.

### GO and pathway enrichment analysis

In this study, the GO terms were defined as being significantly enriched with *P*-values < 0.05 in DAPs and then categorized by three domains: biological process, cellular component and molecular function. At TW-1-M-1 vs. TW-1-1 database, most enriched GO terms in biological process were oxidation reduction (17 DAPs), cellular response to stimulus and stress (26 DAPs), and response to abscisic acid stimulus (7 DAPs), these DAPs should be responsible for seed germination process. The enriched GO terms at TW-1-M-2 vs. TW-1-2 database, which related to seed germination, were response to freezing (7 DAPs), response to water (7 DAPs), seed germination (6 DAPs), seed dormancy (3 DAPs) and dormancy process (2 DAPs). These Go terms belonged to biological process category. In the molecular function category, hydrolase activity (19 DAPs) and catalytic activity (44 DAPs) were the enriched GO terms which might be relative to seed germination. Among TW-1-M-3 vs. TW-1-3 with biological process, the enriched GO terms were response to stress (40 DAPs), response to temperature stimulus (22 DAPs), response to biotic stimulus (18 DAPs), response to cold (15 DAPs), and defense response (15 DAPs). In the molecular function category, oxidoreductase activity (20 DAPs) was the enrichment GO terms. During the whole germination process, the third stage had the most DAPs involved in germination, the second stage contained the mostly catalytic enzyme DAPs. These biological processes might be highly related to seed germination traits. (Additional file 2).

**Fig. 3:**
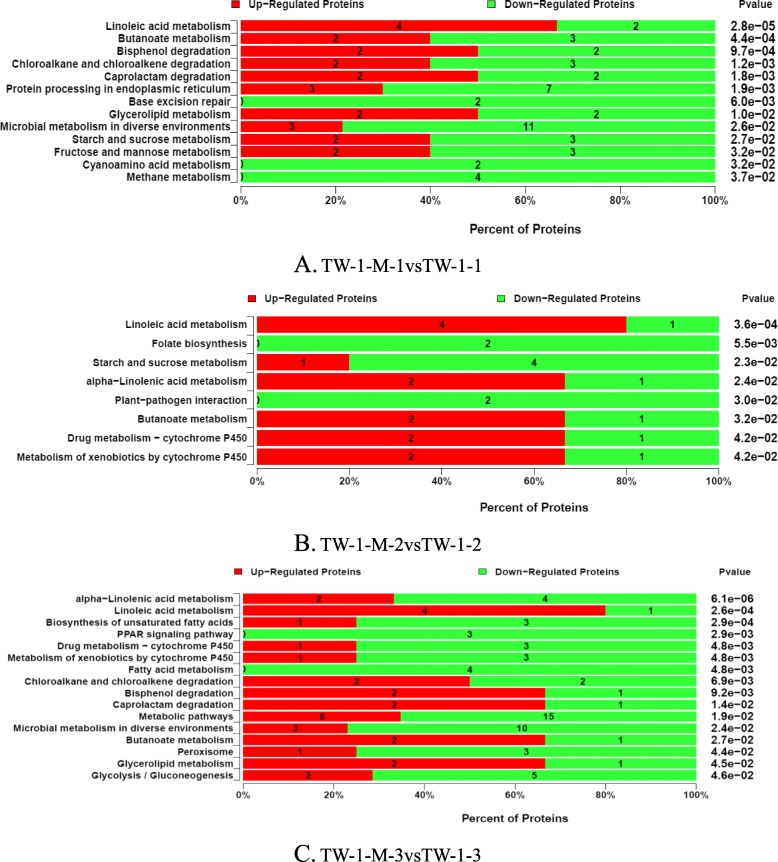
Percentage diagrams illustrating the pathway enrichment analysis. **a** TW-1-M-1vsTW-1-1; **b** TW-1-M-2vsTW-1-2; **c** TW-1-M-3vsTW-1-3

A KEGG pathway enrichment analysis was also done and the pathways were defined as significant DAPs with *P*-values < 0.05. Linoleic acid metabolism was the major enriched pathway which contained 6 DAPs. Butanoate metabolism (5 DAPs), bisphenol degradation (4 DAPs), starch and sucrose metabolism (5 DAPs) and fructose and mannose metabolism (5 DAPs) were also found at the stage of TW-1-M-1 vs. TW-1-1. Linoleic acid metabolism, starch and sucrose metabolism, plant-pathogen interaction and cytochrome P450 pathways were involved at the stage of TW-1-M-2 vs. TW-1-2. These pathways contained 26 DAPs which should be related with seed germination. Linoleic acid metabolism was also the major enriched pathway at TW-1-M-3 vs. TW-1-3 database. Compared with the above two stages, some new pathways were identified, such as glycerolipid metabolism, PPAR signaling pathway and glycolysis/gluconeogenesis. In this stage, 93 DAPs were found and these DAPs might have relationship with germination (Fig. 3, Additional file 3).

### Conjoint analysis of DAPs and DEGs

The combination of transcriptomics and proteomics could provide insights into the environmental and genetic influences on seed germination. Therefore, the correlation between mRNA and protein profiles was further investigated at three stages (TW-1-M-1 vs. TW-1-1, TW-1-M-2 vs. TW-1-2, and TW-1-M-3 vs. TW-1-3). The numbers of genes that correlated at both protein and mRNA levels were 9, 8, and 13 in three stages respectively. These DEGs and DAPs could be divided into three clusters according to their change patterns at the two levels. Cluster I, the patterns of the proteins and genes showed the same trends (3 DEGs/DAPs); Cluster II, the gene change showed the opposite direction with the protein level (8 DEGs/DAPs); Cluster III, the gene level kept unchanged while the protein level showed significant changes (19 DAPs) (Additional file 4). In each stage, there was only one gene which showed the same up/down regulated trends with their proteins, they were *Glyma04g215900* and *Glyma01g095000*; and the third stage (TW-1-M-3 vs. TW-1-3) had the most genes that correlated at both protein and mRNA levels.

Here, two genes (*Glyma01G119600* and *Glyma01G095000*) were identified at all the three stages. According to the *Glyma01G119600* gene, the RNA level was up-regulated but the protein level was down-accumulated at each stage. This gene was annotated as a small hydrophilic plant seed protein, which was functionally classified as responding to stimulus and signaling. For another gene (*Glyma01G095000*), the protein level was up-accumulated at each stage but the mRNA level was up-regulated only at the third stage. These two proteins showed a different regulatory mechanism in the germination environment.

There were six genes identified in each of the two stages. These genes were related to carbohydrate metabolism and responding to stress. The gene (*Glyma06G301500*) related to starch and sucrose metabolism was significantly up-accumulated in the first stage but down-accumulated in the second stage at protein level. However, its RNA levels were down-regulated in both stages. The gene (*LOC100800863*) linked to fructose and mannose metabolism was significantly up-accumulated at protein level in the second and third stages, but its RNA levels were both down-regulated. The disease resistance gene (*Glyma06G319700*) was down-accumulated at protein level in both the second and third stages. Conversely, the RNA levels were up-regulated in these two stages. The gene (*Glyma06G114800*) responsible to stress was significantly up-accumulated in the first stage but down-accumulated in the third stage at protein level.

There were three specific genes in the first stage, which were mainly related with carbohydrate and protein synthesis and metabolism, and they were all down-accumulated at protein level but up-regulated at RNA level. Two specific genes were found in the second stage, and functionally classified in response to stimulus, showed down-accumulated protein level. Only one special gene (*Glyma06G312800*) was up-accumulated at protein level in the third stage.

On the whole, all these genes were related to energy metabolism or response to stress. However, most of these genes from transcriptomic data showed different regulatory levels and directions compared with the proteins from proteomic data. The correlation between the mRNA and protein accumulation profiles may be due to the differential regulation at the mRNA and protein levels.

### Enzyme activity analysis

**Fig. 4:**
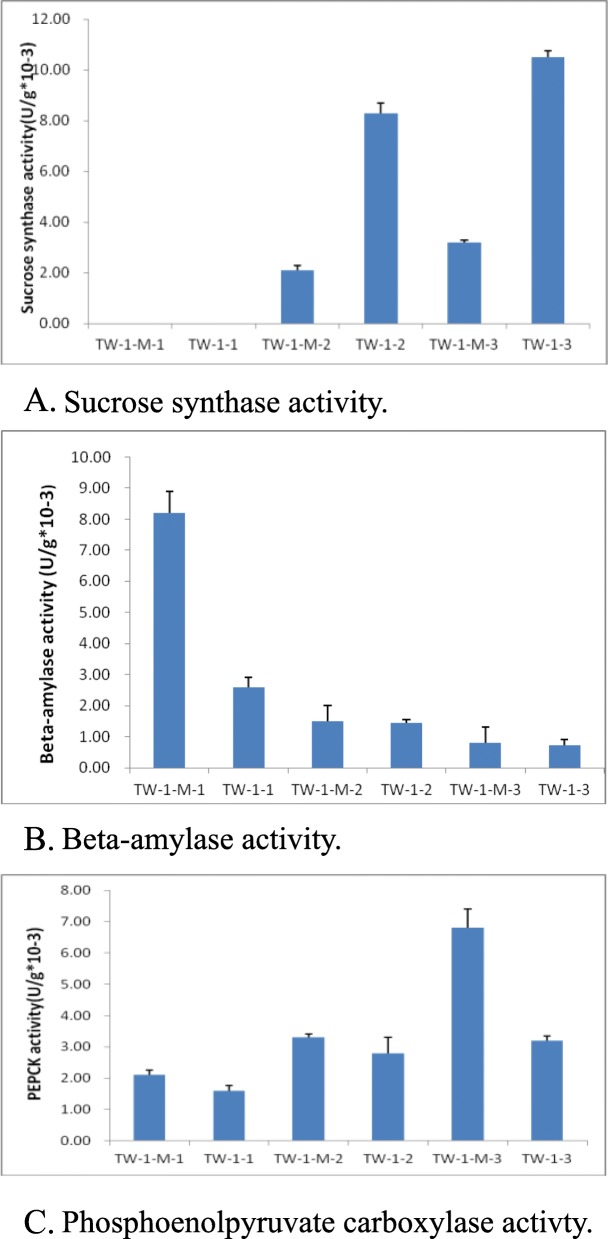
Comparison of enzyme activity between TW-1-M and TW-1 in three germination stages. **a** sucrose synthase activity; **b** beta-amylase activity; **c** phosphoenolpyruvate carboxylase activity. The values shown were means±SD from three biological replicates

To understand enzymes activity of germinating seeds between TW-1 and TW-1-M, the activity of beta-amylase, phosphoenolpyruvate carboxylase and sucrose synthase activity were analyzed. The results showed that the beta-amylase activity in TW-1-M-1 was 3.2 time higher than that of TW-1-1, and 5.6 time than those of TW-1-M-2 and TW-1-2 (Fig. 4). A very low activity of beta-amylase was detected in both TW-1-M-3 and TW-1-3. The activity of sucrose synthase was lower at the second and third stages in TW-1-M compared to TW-1. No activity of sucrose synthase was detected in the first stage of both two mutants. The highest activity of phosphoenolpyruvate carboxylase was found in TW-1-3. All these results were correlated with the iTRAQ results.

## Discussion

It is important to improve seed germination of LPA crops in crop breeding programs. Seed germination is a consequence of interactions among a great number of genes whose expression pattern is genetically pre-programmed but modifiable by environmental influences [14, 17].

### Proteomic analysis between TW-1-M and TW-1

The recent proteomic achievements have broadened knowledge of both biochemical and molecular mechanisms during seed germination [18, 19]. Although many extensive studies have been focused on soybean germination traits [7, 11, 12, 20], the molecular mechanism of germination in low phytate soybean still remains to be further elucidated. To explain the molecular mechanism of LPA seed germination, a proteomic analysis using iTRAQ-based strategy was completed between the two LPA soybean lines which had significantly different germination rates. As a result, 282 DAPs were identified between two mutants at three stages, 80 were down-accumulated and 202 were up-accumulated. The analysis showed that these DAPs were mainly annotated in biological and abiotic stress and energy metabolism process functional groups. The KEGG analysis showed that these DAPs mainly participate in carbohydrate (sucrose and fatty acid), energy metabolism and anti-stress pathways. These results were partly consistent with the previous reports from other plants, such as wheat [15], rice [21], garden pea [22] and Arabidopsis [23]. These reports suggested that the genes related to plant hormones and reactive oxygen species might play key roles during seed germination.

### DAPs involved in protein biosynthesis and metabolism

Seed germination process is a complicated process in higher plants, which involves the seed imbibition, seed germination and seeding growth. The initial nutrients required for the process is transformed from the reserve substances in seed [24]. The protein and fat are the main storage materials during germination in soybean. In our study, 10 DAPs were annotated as the KEGG pathway of protein processing in endoplasmic reticulum during the first germination phase. Among these proteins, a soybean seed maturation protein cDNA *GmPM31* was found, encoding a class I low-molecular-weight heat shock protein. The *GmPM31* protein might be seed-maturation specific, and encode the protein localized in the cytoplasm [25], which was up-accumulated in TW-1-M-1.

The protein disulfide isomerase (PDI) encodes an enzyme in the endoplasmic reticulum in eukaryotes and plays a primary role as a supplier of disulfide bonds in nascent proteins for oxidative folding on the endoplasmic membrane [26]. The GmPDIL (*Glyma06G114800*) was up-accumulated in TW-1-1 whereas the other GmPDI (*Glyma04G215900*) was up-accumulated in TW-1-M-1, suggesting a separate role in the conformational stability and function of proteins. In addition, the differential accumulation of these proteins might be associated with the activity mechanism of seed germination under salt stress [26, 27].

The cyanoamino acid metabolism pathway contained two DAPs, one of them was serine hydroxymethyltransferase (*Glyma05G152100*), which was down-accumulated in TW-1-M-1. The serine hydroxymethyltransferase (*SHMT*, *EC 2.1.2.1*) is a ubiquitous enzyme essential for cellular one-carbon metabolism present in all organisms, catalyzing the reversible conversions of serine and glycine. Therefore, they provide one-carbon units for a series of biosynthetic processes, such as methionine, thymidylate, and purine syntheses [28–31].

### DAPs involved in lipid biosynthesis and metabolism

The mature soybean seed have crude fat content up to 20%, they mainly provide energy for seed germination [32]. Linoleic acid metabolism pathway was the most common pathway during the soybean seed germination stage. The Lipoxygenases, catalyzing the dioxygenation of polyunsaturated fatty acids, were the most common, clearest proteins and always up-accumulated in TW-1-M during the germination stage. The previous study showed that the *OsLOX2* over-expressing rice seeds showed lower germination rates after aging treatments, but they could germinate faster under normal conditions [33]. Furthermore, a high activity of lipoxygenase could provide more energy for seed germination. In addition, the phosphoenolpyruvate carboxylase was down-accumulated in TW-1-M-3, which has previously been reported to contribute in the synthesis and metabolism of oil [34].

### DAPs involved in carbohydrate metabolism

Although the carbohydrate content is much lower than that of protein and crude fat in soybean seed, the carbohydrate can be utilized more easily in metabolism due to its simple molecular structure [5, 7]. During seed germination and early seedling establishment, the energy supporting these biological processes comes primarily from carbon reserves stored in the seed itself, with sucrose and fructose being the main forms of these carbon reserves [35]. In this study, most of the pathways that contained DAPs were related with energy and carbohydrate metabolism, such as starch and sucrose metabolism pathway, fructose and mannose metabolism pathway and glycolysis/gluconeogenesis pathway. The conjoint analysis also suggested that some shared DEGs/DAPs were associated with the pathways related to carbohydrate metabolism. These proteins might provide energy, translate signaling or be involved in the anti-oxidative process. The enhanced carbohydrate metabolism would accelerate reserve hydrolysis and impair protein biosynthesis during seed germination [36, 37].

Many DAPs were identified related to carbohydrate metabolism, such as alpha-1,4 glucan phosphorylase (representing an amenable system for the synthesis of extended amylose chains up to 80 or more residues whilst retaining control over the chain length) [38], glucose-6-phosphate isomerase (catalyzing the reversible isomerization of glucose-6-phosphate and fructose-6-phosphate, a reaction that also immediately precedes sucrose biosynthesis in plants) [39], pyrophosphate-fructose 6-phosphate 1-phosphotransferase (catalyzing the reversible interconversion between fructose-6-phosphate and fructose-1,6-bisphosphate, a rate-limiting step in the regulation of the primary carbohydrate metabolic flux toward glycolysis or gluconeogenesis) [40], sucrose synthase (catalyzing the UDP-dependent cleavage of sucrose into UDP-glucose and fructose) [41], and beta-amylase (cleaving α-1,4 glucosidic bonds at the non-reducing end of polyglucan chains to produce maltose) [42] . These genes with up/down-regulation made a contribution to seed germination process. The transcript profiling of these LPA mutants also suggested some highly expressed and enriched genes related to carbohydrate biosynthesis and metabolism pathways [14]. These results showed that carbohydrate metabolism had a close relationship with germination trait of LPA soybean mutants. These findings were in agreement with the results from other plants [43, 44]. Furthermore, we found that these DAPs had no special regulation trend in the TW-1-M with high germination rate, which means that each protein had a separate regulating way for seed germination process.

### DAPs response to stress

Four DAPs were annotated in the KEGG pathway of peroxisome at last germination stage (TW-1-M-3 vs. TW-1-3). Among them, two proteins were annotated as peroxisomal 3-ketoacy1-CoA, which was required in jasmonic acid (JA) biosynthesis. These two proteins were all down-accumulated in TW-1-M-3. The previous report showed that the JA synthesis is essential for resistance to male reproductive function and chewing insects, and thus different types of biotic stress might induce JA synthesis via distinct enzymatic routes [45].

The Cysteine proteinase inhibitor (CPI) protein level was significantly down-accumulated in TW-1-M-1. The study showed that the CPIs involved in plant response to environmental stresses exhibited different expression patterns [46]. For example, the expression of *AtCYS6*, induced by the germination inhibitory phytohormone ABA, inhibited the cysteine proteinases activity to regulate seed germination and seedling growth [47]. The studies also indicated that cystatins could be response to cold and drought stress during plant development [48, 49].

The level of non-specific lipid-transfer protein (*NsLTP*) that facilitated the transportation of fatty acids, phospholipids, and steroids between membranes was down in TW-1-M-3. The study showed that the overexpression of the pepper *CALTP1* gene increased its tolerance to NaCl and drought stresses in Arabidopsis [50]. Another study showed that *OsLTPL36*, a seed specific lipid transporter, might play an important role to regulate seed development and germination [51].

We also found many DAPs annotated as uncharacterized protein. The functions of most of them could be divided into the response to stimulus. These results showed that germination process of two LPA mutants was similar with some processes induced by biological or abiotic stress. The TW-1-M mutant with a high germination rate would have more regulatory mechanisms to answer the lowering phytate level in soybean seeds.

### DAPs response to reactive oxygen species and plant hormones

The comparative transcript analysis of TW-1-M and TW-1 indicated that the expression diversification of reactive oxygen species-related genes and plant hormone-related genes may be strongly responsible for the successful germination in TW-1-M [14]. In our study, the DAPs were also identified related to reactive oxygen species and plant hormones.

Some DAPs were identified to maintain oxidative balances and reduce oxidative damage to a wide range of cellular components, such as DNAs, proteins and lipids [52, 53]. Among these DAPs, three proteins were found, including acyl-coenzyme A oxidase, peroxisomal-3-ketoacyl-CoA thiolase and glutathione-s-transferase. These enzymes all play a very important role in lipid transfer and metabolism. In addition, the ascorbate peroxidase, with the high accumulation in TW-1, would play a role in the regulation of the oxidative state and maintaining seed vigor during the early germination stage [54]. The Cytochrome P450 family, found in both TW-1 and TW-1-M with different accumulation trends, is a large group that mediate a diverse array of oxidative reactions [55]. Some DAPs related to flavone metabolism were identified, such as isoflavone reductase. These proteins are involved in isoflavone biosynthesis and isoflavone is considered as an anti-oxidative compound in soybean seeds.

Seed germination is controlled by intrinsic and environmental cues, which are mainly regulated by plant hormones, such as gibberellin (GA) and abscisic acid (ABA) [16]. However, no DAPs were found related to GA and ethylene in this study. This may be due to the detection limit of large-scale proteome analysis. We found three DAPs involved in ABA metabolism and signal transduction, such as *AtCYS6* that was also detected in DAPs response to stress. The accumulation levels of these three proteins were lower in TW-1-M than TW-1, which could be responsible for TW-1-M’s high germination. This finding was consistent with the transcript profiling results [14].

### Conjoint analysis of DAP/DEG

The DEGs from transcriptome data and the DAPs from proteome data were combined for samples from three different stages to better understand the differences. The disagreement should be reduced in the mRNA-protein correlation since both the transcriptome and the proteome data were obtained from the same sample. The quantified proteins involved in anti-stress process and energy metabolism were most explored, as well as many DEGs related to abiotic and biotic stresses in transcript profiling. However, their regulatory directions were almost opposite. Furthermore, the mRNA and protein changes of some genes were not even correlated. Therefore, the protein accumulation might depend on multiple metabolic and regulatory pathways in biological systems [56].

By the conjoint analysis of the DAPs and DEGs, 30 shared DAPs/DEGs were identified with different expression patterns among three germination stages in two LPA soybean mutants. Although the numbers of genes correlated at both mRNA and protein levels were limited, we were still interested with those mRNAs and proteins that shared a common direction. The results showed directional correlation for 3 mRNAs/proteins while 8 mRNAs/proteins were inversely correlated. The regulatory mechanisms of the genome and proteome are so complex that both turnover and stability of mRNA levels are important for the translation of mRNA into protein [57, 58]. For example, the gene (*Glyma01G119600*), annotated as a small hydrophilic plant seed protein, exhibited an increased level of mRNA but a decreased level of protein, which suggested that there might be an increased protein degradation leading to induced transcription. Future studies aimed at examining the specific genes at both RNA and protein levels would further provide insights into the germination process of LPA crops.

## Conclusions

The protein accumulation profiling would offer a substantial contribution to investigate the germination mechanism in LPA mutants. Here, 282 DAPs were identified between these two mutants at three stages. Among them, 80 DAPs were down-accumulated and 202 were up-accumulated. Bioinformatic analysis showed that the identified proteins were related to functional categories of oxidation reduction, response to stimulus and stress, dormancy and germination processes and catalytic activity. KEGG analysis showed that these DAPs were mainly related with energy metabolism, such as carbohydrate metabolism and fat metabolism, and anti-stress pathways. In addition, 30 shared DAPs/DEGs were identified by the conjoint analysis of the DAPs and DEGs with different expression pattern among three germination stages in two LPA soybean mutants. These proteins included plant seed protein, beta-amylase, protein disulfide-isomerase, disease resistance protein, pyrophosphate-fructose 6-phosphate 1-phosphotransferase, cysteine proteinase inhibitor, non-specific lipid-transfer protein, phosphoenolpyruvate carboxylase and acyl-coenzyme A oxidase. The TW-1-M and TW-1 displayed a number of DAPs that participated in seed germination. The differential accumulation of these proteins would make a high contribution to the germination trait of LPA mutants. The accumulation diversification of reactive oxygen species-related and plant hormone-related proteins might strongly contribute to the successful germination in TW-1-M. These findings would help us further explore the mechanisms of seed germination in LPA crops.

## Methods

### Plant materials

Two LPA soybean mutants, Gm-lpa-TW-1-M (TW-1-M) and Gm-lpa-TW-1 (TW-1), that were developed in our lab were planted and then used to evaluate the protein accumulation. The TW-1 was generated using gamma irradiation from the commercial variety, Taiwan-75. The TW-1-M was selected as a natural mutant from the TW-1 mutant. The TW-1-M and TW-1 both had a deletion of 2 bp in the *GmMIPS1* gene at the same mutation site with a comparable level of phytic acid content. Seed samples were collected in 2016 at the research fields in Hangzhou, China.

To better understand and compare the accumulation differences between the TW-1-M and TW-1 mutants at seed germination, three individual stages were selected for further analysis, including 1) the imbibed seed stage that corresponds to seeds soaked for about 24 h, named TW-1-M-1 and TW-1-1, 2) the metabolism reactivation phase between seed imbibition and radicle emergence that corresponds to seeds soaked for about 30 h, named TW-1-M-2 and TW-1-2, and 3) the emergence of primary roots with 1 mm in length that corresponds to seeds soaked for about 36 h, named TW-1-M-3 and TW-1-3 [14]. To minimize environmental effects, three sets of parallel samples collected at each stage were used to extract proteins for biological replicates.

### Germination experiment

To evaluate the differences in germination ability of these two LPA lines, germination experiments were conducted with two different treatments that were warm germination and accelerated aging, respectively following the procedure described by Yuan et al. [13]. For each treatment, 100 seeds were used for each of the two LPA mutants (TW-1-M and TW-1) and their wild type parent (Twaiwan-75) with three replicates.

### Protein preparation

About 0.5 g of frozen seed samples were pulverized to powder in liquid nitrogen with a mortar and pestle. The fine powder was suspended with 200 μl dissolution buffer (50 mM triethyl ammonium bicarbonate (TEAB), 8 M Urea, pH 8). The sample was broken by the ultrasonic wave for 15 min, and then centrifuged at 12,000 r/min for 10 min at 4 °C. The supernatant was mixed with 800 μl ice-cold acetone that contained 10 mM Dithiothreitol (DTT) for 2 h at 4 °C. The mixture was centrifuged at 12,000 r/min for 10 min at 4 °C. The protein precipitate was washed three times using ice-cold acetone, and then dried for 30 min by centrifugal vacuum evaporation. The dried precipitate was resolubilized with 100 μl dissolution buffer. The total protein concentration was assayed using a BCA protein assay kit (Thermo Scientific, USA). One hundred microgram of each protein sample was dissolved in dissolution buffer to a total volume of 100 μl, and then diluted with 500 μl 50 mM NH_4_HCO_3_. After reduction and alkylation, 2 μg of trypsin was added, and then incubated for digestion at 37 °C overnight. The digestion was stopped by adding trifluoroacetic acid (FA) to a final concentration of 0.1%. The peptides were purified on Strata–X C18 pillar (Phenomenex, USA) and dried by centrifugal vacuum evaporation. The dried peptides were redissolved with 20 μl 0.5 M TEAB and then labeled with iTRAQ Reagent-8PLEX Multiplex kit (AB Sciex, UK). The samples of TW-1-1, TW-1-2, TW-1-3, TW-1-M-1, TW-1-M-2 and TW-1-M-3 were labeled with 113-tag, 114-tag, 115-tag, 116-tag, 117-tag and 118-tag, respectively. Next, the labeled samples were fractionated using high performance liquid chromatography system (Thermo DINOEX Ultimate 3000 BioRS) using a Durashell C18 (5 um, 100 A, 4.6 × 250 mm). At last, 12 fractions were collected.

### LC-MS/MS analysis

The labeled samples of TW-1-1, TW-1-2, TW-1-3, TW-1-M-1, TW-1-M-2 and TW-1-M-3 were mixed with equal amount, and then fractionated on a Durashell C18 column (4.6 × 250 mm, 5 μM, 100 A) using DINOEX Ultimate 3000 BioRS HPLC system (Thermo Scientific, USA). Data were collected on a TripleTOF 5600 plus system (AB SCIEX, UK) using a 90-min gradient from 2 to 30% with buffer A (0.1% (v/v) formic acid, 5% (v/v) acetonitrile) and buffer B (0.1% (v/v) formic acid, 95% (v/v) acetonitrile). A parent-ion scan was performed over the range of 350–1500 m/z for 250 ms and MS/MS spectra were collected on the 20 most intense parent ions with charge state 2–5 in the range 50–2000 m/z for 100 ms. The samples of TW-1-1, TW-1-2, TW-1-3, TW-1-M-1, TW-1-M^− 2^ and TW-1-M-3 were analyzed in triplicate and each replicate of the six samples was run in a batch (LC-BIO TECH, Hangzhou, China).

### Data interpretation, analysis of differentially accumulated proteins (DAPs)

The original MS/MS data were searched with ProteinPilot software v4.5 with the parameters as follows: iTRAQ quantification; trypsin digestion; cysteine modified with iodoacetamide; Background Correction and Bias Correction checked; Biological modifications as ID focus; Uniprot *Glycine max* as database. The false discovery rate (FDR) was calculated with an automatic decoy database search strategy using the PSPEP (Proteomics System Performance Evaluation Pipeline Software, integrated in the ProteinPilot Software) algorithm. The proteins identified with unused score more than 1.3 (confidence level > 95%) and at least one unique peptide were considered for further analysis. For protein abundance ratios after normalization, a *P*-value less than 0.05 and 1.2-fold change was applied as the threshold to compare significant changes. The e-value was set as less than 1e-5. The best hit for each query was selected for GO term matching. The GO term matching was performed with blast2go v4.5 pipeline5. The Clusters of Orthologous Groups (COG) were used to annotate gene functions. We also conducted hypergeometric test to analyze GO enrichment and KEGG pathway enrichment.

### Conjoint analysis of DEGs and DAPs

The accession numbers were collected from the transcriptomic dataset, and then compared with the annotated iTRAQ database to conjoint analysis of DAPs and DEGs [59]. The transcriptome sequencing results were employed for coding sequence. Then the accession numbers from the proteomics dataset were also used to search the same coding sequence database. At last, conjoint dataset of DPAs and DEGs was obtained. We set ∣FC∣ > 1.2, *P* < 0.05 and∣FC∣ > 2, P < 0.05 as the threshold to extract DEGs and DAPs subsets.

### Enzyme activity analysis

Beta-amylase, phosphoenolpyruvate carboxylase, and sucrose synthase activity were measured by beta-amylase (AMS) test kit, phosphoenolpyruvate carboxylase (PEPC) test kit, and sucrose synthase assay kit, respectively (Jiancheng, Nanjing, China). The detective methods were according to their respective protocols in kits with three biological replicates. For each biological replicate, there were three technical replicates.

## Supplementary information

**Additional file 1.** The mainly regulatory pathways (without directions) with the most protein numbers in the three stages.

**Additional file 2.** The enriched GO terms in the three stages.

**Additional file 3.** The enriched KEGG pathways in the three stages.

**Additional file 4.** The correlations between mRNA and protein in the three stages.

**Additional file 5: Figure S1.** Venn diagrams showing the overlapping of identified proteins in the three batches.

**Additional file 6: Figure S2.** Functional categorization of DAPs during the seed germination stage. A: Functional categorization of DAPs between TW-1-M-1 and TW-1-1. B: Functional categorization of DAPs between TW-1-M-2 and TW-1-2. C: Functional categorization of DAPs between TW-1-M-3 and TW-1-3.

## Data Availability

The mass spectrometry proteomics data have been deposited to the ProteomeXchange Consortium (http://proteomecentral.proteomexchange.org) via the iProX partner repository [60] with the dataset identifier PXD012808.
