# Supplementary figures and images for: Quantitative proteomic analyses of two soybean low phytic acid mutants to identify the genes associated with seed field emergence
Source: BMC Plant Biol. 2019 Dec 19;19:569. doi: 10.1186/s12870-019-2201-4 (PMC6921446; doi:10.1186/s12870-019-2201-4)

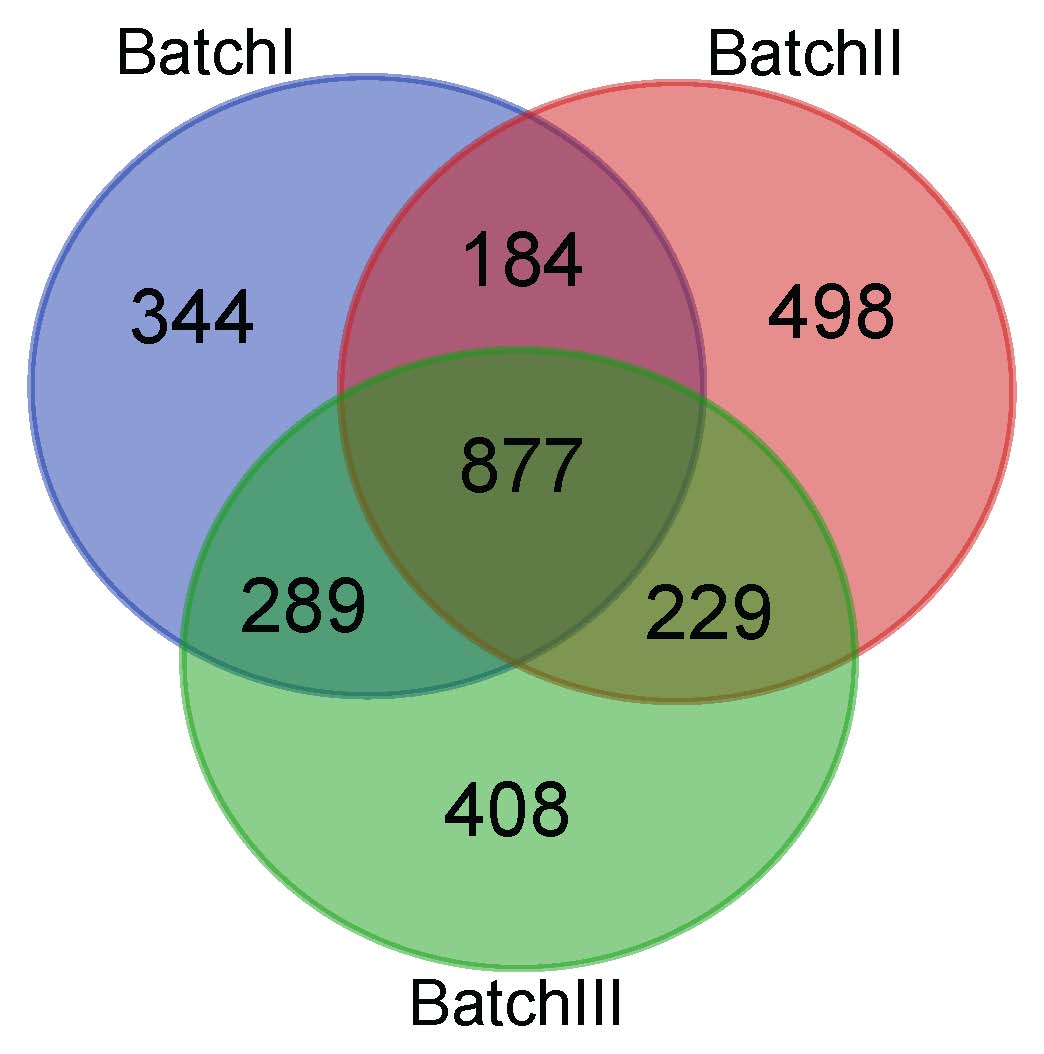


Fig. S1. Venn diagrams showing the overlapping of identified proteins in the three batches.


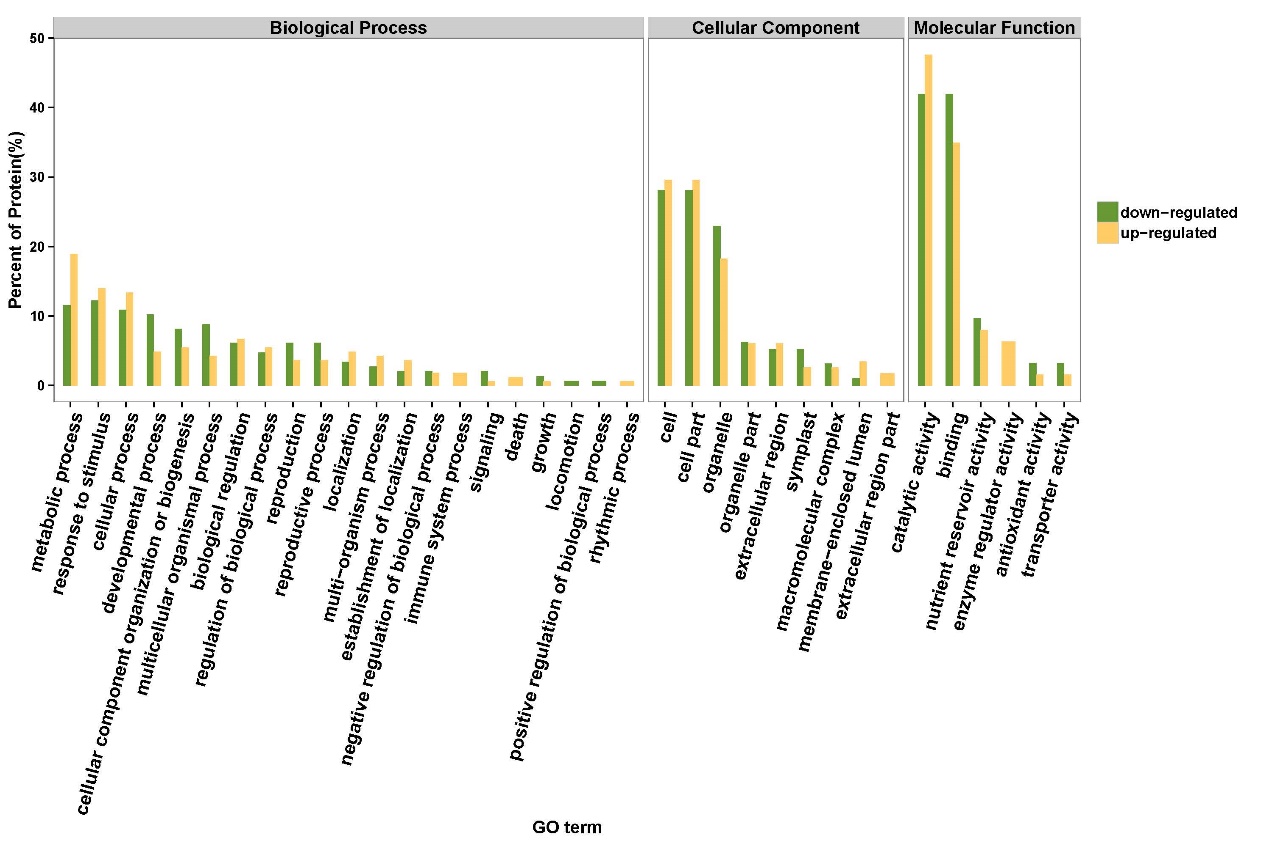

Supplement: Supplementary file 5 — Additional file 5: Figure S1. Venn diagrams showing the overlapping of identified proteins in the three batches. [file 12870_2019_2201_MOESM5_ESM.docx]
