# Supplementary material for: Quantitative proteomic analyses of two soybean low phytic acid mutants to identify the genes associated with seed field emergence
Source: BMC Plant Biol. 2019 Dec 19;19:569. doi: 10.1186/s12870-019-2201-4 (PMC6921446; doi:10.1186/s12870-019-2201-4)
Supplement: Supplementary file 6 — Additional file 6: Figure S2. Functional categorization of DAPs during the seed germination stage. A: Functional categorization of DAPs between TW-1-M-1 and TW-1-1. B: Functional categorization of DAPs between TW-1-M-2 and TW-1-2. C: Functional categorization of DAPs between TW-1-M-3 and TW-1-3. [file 12870_2019_2201_MOESM6_ESM.docx]

Fig. S2A. Functional categorization of DAPs between TW-1-M-1 and TW-1-1.


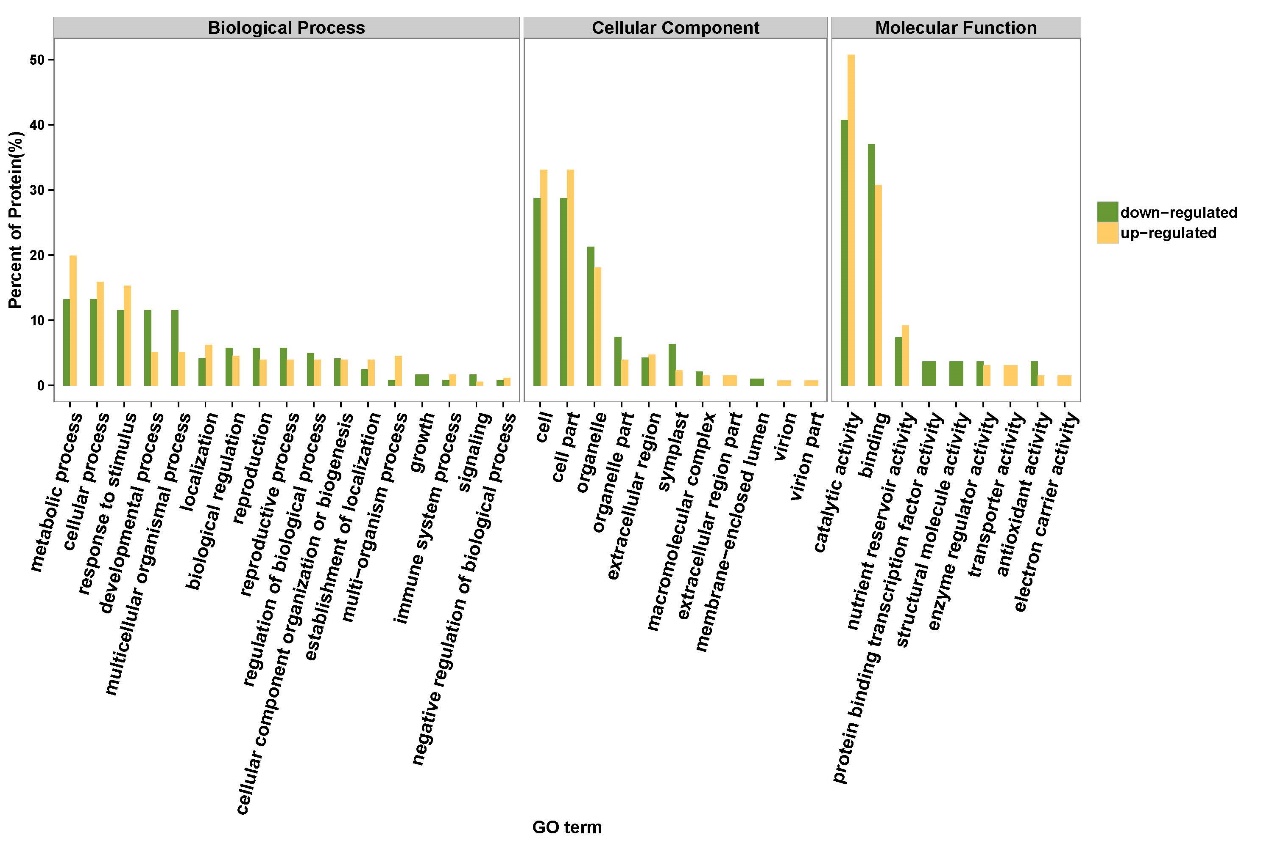


Fig. S2B. Functional categorization of DAPs between TW-1-M-2 and TW-1-2.


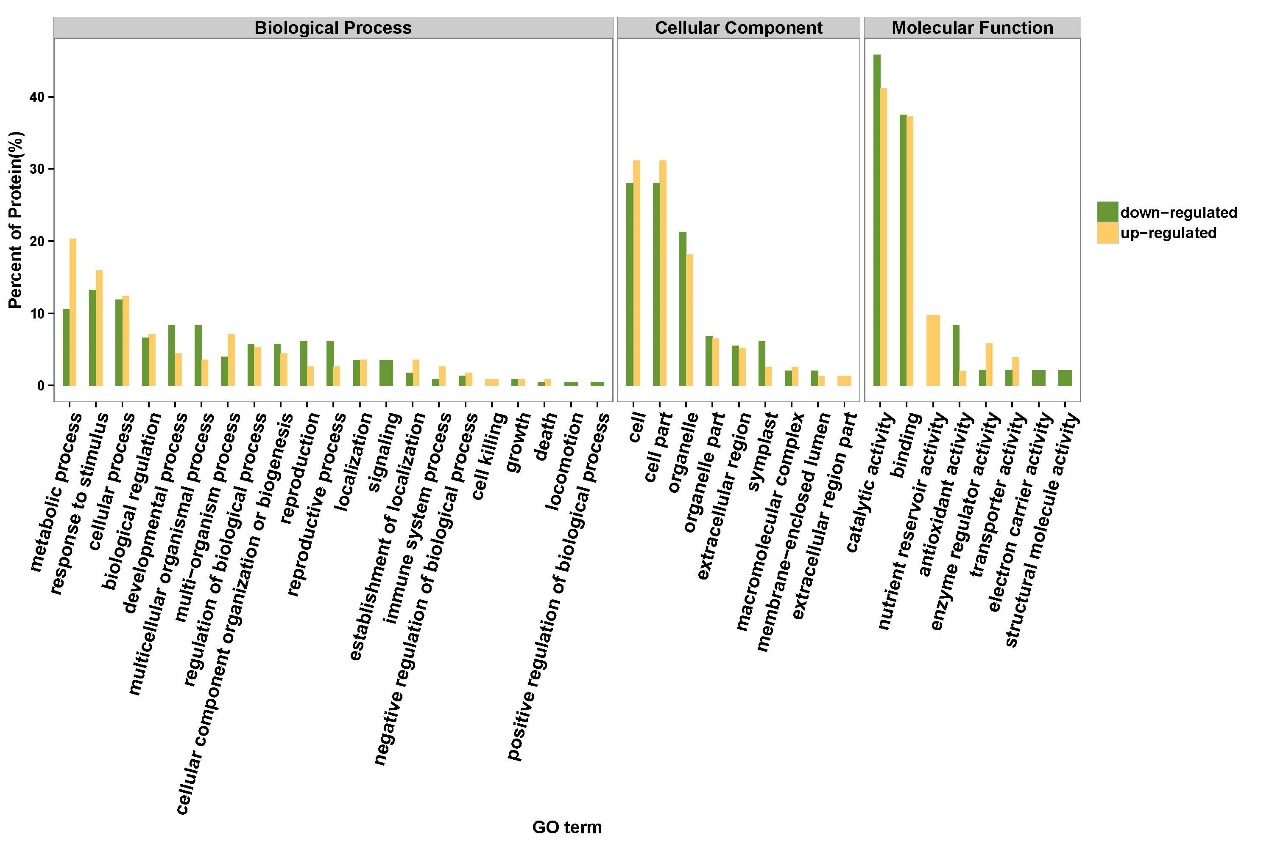


Fig. S2C. Functional categorization of DAPs between TW-1-M-3 and TW-1-3.

Fig. S2. Functional categorization of DAPs during the seed germination stage. A. Functional categorization of DAPs between TW-1-M-1 and TW-1-1. B. Functional categorization of DAPs between TW-1-M-2 and TW-1-2. C. Functional categorization of DAPs between TW-1-M-3 and TW-1-3.
